# LHX2 Mediates the FGF-to-SHH Regulatory Loop during Limb Development

**DOI:** 10.3390/jdb6020013

**Published:** 2018-06-15

**Authors:** Billy A. Watson, Jennifer M. Feenstra, Jonathan M. Van Arsdale, Karndeep S. Rai-Bhatti, Diana J. H. Kim, Ashley S. Coggins, Gennaya L. Mattison, Stephen Yoo, Eric D. Steinman, Charmaine U. Pira, Brendan R. Gongol, Kerby C. Oberg

**Affiliations:** 1 Department of Pathology and Human Anatomy, School of Medicine, Loma Linda University, Loma Linda, CA 92354, USA; bawatson@llu.edu (B.A.W.); jennifer.feenstra@ki.se (J.M.F.); Jonvan83@gmail.com (J.M.V.A.); kraib001@ucr.edu (K.S.R.-B.); djhkim@llu.edu (D.J.H.K.); ashley.coggins.1@gmail.com (A.S.C.); gennaya.mattison@gmail.com (G.L.M.); Yoomanchu@gmail.com (S.Y.); esteinman@gmail.com (E.D.S.); cpira@llu.edu (C.U.P.); 2 Division of Microbiology and Molecular Genetics, Department of Basic Sciences, School of Medicine, Loma Linda University, Loma Linda, CA 92354, USA; 3 Department of Cardiopulmonary Sciences, School of Allied Health Professions, Loma Linda University, Loma Linda, CA 92354, USA; bgongol@llu.edu

**Keywords:** limb development, fibroblast growth factor (FGF), sonic hedgehog (SHH), LIM Homeobox 2 (LHX2)

## Abstract

During limb development, fibroblast growth factors (Fgfs) govern proximal–distal outgrowth and patterning. FGFs also synchronize developmental patterning between the proximal–distal and anterior–posterior axes by maintaining Sonic hedgehog (Shh) expression in cells of the zone of polarizing activity (ZPA) in the distal posterior mesoderm. Shh, in turn, maintains Fgfs in the apical ectodermal ridge (AER) that caps the distal tip of the limb bud. Crosstalk between Fgf and Shh signaling is critical for patterned limb development, but the mechanisms underlying this feedback loop are not well-characterized. Implantation of Fgf beads in the proximal posterior limb bud can maintain *SHH* expression in the former ZPA domain (evident 3 h after application), while prolonged exposure (24 h) can induce *SHH* outside of this domain. Although temporally and spatially disparate, comparative analysis of transcriptome data from these different populations accentuated genes involved in SHH regulation. Comparative analysis identified 25 candidates common to both treatments, with eight linked to *SHH* expression or function. Furthermore, we demonstrated that LHX2, a LIM Homeodomain transcription factor, is an intermediate in the FGF-mediated regulation of SHH. Our data suggest that LHX2 acts as a competency factor maintaining distal posterior *SHH* expression subjacent to the AER.

## 1. Introduction

Limb development generates a structure that has asymmetry along three coordinate axes: proximal–distal (PD), anterior–posterior (AP), and dorsal–ventral (DV). Each axis has its own signaling center that mediates patterning. The apical ectodermal ridge (AER), a thickening of ectoderm located along the distal rim of the limb, is a signaling center from which secreted fibroblast growth factors (Fgfs) regulate PD patterning and outgrowth. Sonic hedgehog (Shh) is secreted from a cluster of cells in the distal posterior limb bud mesoderm called the zone of polarizing activity (ZPA) and regulates AP expansion and patterning. The dorsal ectoderm secretes Wnt7a, which dorsalizes the developing limb. During development, crosstalk among these axes is required to coordinate proper limb patterning [1].

Along the PD and AP axes, development is coordinated through crosstalk between Fgf and Shh in a reciprocal feedback loop [2,3]. The mechanism by which Shh maintains Fgf in the AER is relatively well-characterized and includes Shh directing interactions between Formin, Gremlin, and Bone morphogenic proteins (Bmps) [4,5,6]. In the absence of Shh, the AER regresses and posterior elements of the developing limb are lost [7,8]. In contrast, the mechanism by which AER-secreted Fgfs regulate *Shh* is less clear. As the limb bud elongates, the ZPA (and *Shh* expression) persists distally subjacent to the AER. *Shh* expression in the more proximal posterior mesodermal cells, which we refer to as the former ZPA domain, wanes as the cells move beyond the influence of the AER-Fgfs [9,10]. 

**Figure 1:**
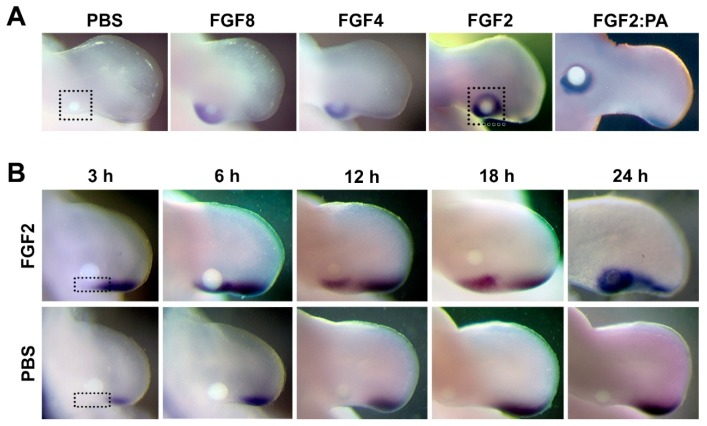
FGF has the capacity to maintain or ectopically induce SHH expression. (**A**) SHH expression following 24 h of exposure to FGF8, FGF4, or FGF2-soaked beads implanted into the posterior HH23 chicken wing mesoderm proximal to the ZPA (500 μm from the AER). Ectopic SHH expression is present around the FGF bead, but not in the PBS (vehicle control). This ectopic expression was noted in all embryos tested (n = 3 for each FGF). The expression was present in cells of the former ZPA domain (along the posterior margin) and in cells that likely lacked prior ZPA exposure (anterior aspect of the bead). Induction of non-ZPA cells was accentuated when initial bead placement was more proximal (600 μm from the AER) and anterior (500 μm from posterior edge) (FGF2:PA). (**B**) Persistent SHH expression is evident in former ZPA domains over a 3–24 h time course post-FGF bead implant. In addition, robust ectopic SHH expression is present around the FGF bead at 24 h (top panel) as compared to the PBS control (bottom panel). For each time point, 5 embryos were assayed for response to FGF, while 3 embryos were for expression in the presence of PBS (vehicle control). Two independent experiments were used to collect embryos, and the pattern of ectopic SHH expression was consistent in all time-matched embryos. Tissue harvested for transcriptome analysis and RT-qPCR validation is indicated by black dotted lines.

In the chicken, FGF2 [11,12], FGF4 [2,13,14], or FGF8 [15,16] is sufficient to recover *SHH* expression in the absence of the AER. When applied to the mid-posterior half of the limb bud, nearly 24 h is required to upregulate *SHH* in cells outside the ZPA or former ZPA domain (Figure 1A). Interestingly, in cells of the former ZPA domain (proximal posterior limb margin), *SHH* could be maintained in the presence of proximal FGF, evident within 3 h of exposure. The former ZPA domain and the non-ZPA domain differ in context (maintenance versus induction, respectively); nevertheless, we suspected that the final pathway to *SHH* expression is common in both domains. 

*FGF2* is endogenously expressed in the chicken [11,17] and human AER [18], has broader effects than FGF4 and FGF8 [19], and interacts with greater affinity to the primary ZPA-related Fgf receptor (FGFr1cIII) [20,21]. Thus, we applied FGF2-laden beads to chicken limb buds to generate ZPA domain and the non-ZPA domain transcriptomes. Using comparative analysis, we identified genes that were common to both transcriptomes, genes we suspected would accentuate the FGF-to-SHH pathway. From our analysis, we were able to identify a number of candidates in the FGF-to-SHH pathway and confirm a role for LIM Homeobox 2 (LHX2) as an intermediate in FGF-regulated *SHH* expression.

## 2. Materials and Methods

### 2.1. FGF Bead Implants, WNT5a Cell Implants, and Harvesting Embryos

Heparin acrylic beads (H5263 Sigma, St. Louis, MO, USA), about 150 μm in diameter, were soaked in 4 μL of 0.5 mg/mL recombinant human FGF2 (233-FB), FGF4 (235-F4), or FGF8 (423-F8) (R&D Systems, Minneapolis, MN, USA) overnight at 4 °C or for at least 1 h at room temperature. For the control samples, the heparin acrylic beads were soaked in 4 μL 1× phosphate-buffered saline (PBS) before implantation. Using tungsten needle and forceps, FGF2 or PBS-soaked beads were implanted (*in ovo*) into the posterior forelimb bud mesoderm of 23 Hamburger–Hamilton stage (HH) chicken embryos about 500 μm from the distal tip. Cells secreting Wnt5a (CRL-2814, ATCC^®^, Manassas, VA, USA) were grown to confluence and then stained with neutral red. Strips of Wnt5a-secreting cells were then harvested and implanted into the posterior margin of HH23 right limb bud mesoderm as previously described [22]. Embryos were incubated at 39 °C in a humidified chamber for the experimental time (3–24 h) and quickly harvested in PBS. Tissue from embryos for transcriptome and RT-qPCR analyses was removed using a tungsten needle, flash frozen using liquid nitrogen, and stored at −80 °C until further processing. Embryos for whole-mount in situ hybridization (WMISH) were fixed in MEMFA (1 M MOPS, 20 mM EGTA, 10 mM MgSO4, 38% formaldehyde) overnight at 4 °C as described previously [23], then stored in 90% methanol at −20 °C until further processing.

### 2.2. RNA-Seq Analysis

After 3 h of incubation, the tissue directly posterior to the FGF2 or PBS bead was extracted, and RNA was isolated using the RNeasy Plus Micro Kit (Qiagen, Valencia, CA, USA). Three independent experiments were conducted and RNA within each sample group (FGF or PBS) from each experiment was pooled to decrease genetic variability (n = 15–20 embryos per sample). RNA-seq was conducted by Norris Comprehensive Cancer Center, University of Southern California. RNA-seq differential gene expression and statistical analysis was conducted in R coding language with the systempipeR package tools. Reads were aligned with Hisat2 alignment algorithm to release-87 of *Gallus gallus* genome build 5. Fold changes were calculated, and statistical comparisons made with EdgeR. Genes exhibiting differential expression between FGF2- and PBS-treated embryos with *p* < 0.05 were included in further analyses. RNA-seq data were submitted to Gene Expression Omnibus (http://www.ncbi.nlm.nih.gov/geo/) [24] and can be located under series accession number GES114663.

### 2.3. DNA Microarray Analysis

RNA was isolated from tissue surrounding an FGF2 or PBS bead after 24 h of incubation using the RNeasy kit (Qiagen). The RNA from 6 embryos was pooled for each treatment group to decrease genetic variability, and the experiment was repeated 3 times on separate days. Microarray analysis was performed by University of California at Irvine Genomics High Throughput Facility as previously described [23]. Samples were hybridized to the Affymetrix GeneChip^®^ Chicken Genome Array (ThermoFisher Scientific, Chino, CA, USA). The data was normalized using Robust Multi-array Averaging (RMA) and analyzed using the Comprehensive R and Bioconductor-based web service for microarray data analysis. Only genes exhibiting differential expression between FGF- and PBS-treated embryos with *p* < 0.05 were included in further analyses. Microarray data were submitted to Gene Expression Omnibus and can be located under series accession number GES114663. 

### 2.4. Gene Ontology

Differentially expressed genes at 3 and 24 h post-FGF bead implantation were classified according to gene ontology biological pathways using Ingenuity Pathway Analysis (IPA) software and database (Qiagen).

### 2.5. Gene Expression Analysis via Whole-Mount In Situ Hybridization (WMISH)

To validate differential gene expression in response to 3 and 24 h of FGF treatment, digoxigenin-labeled probes were generated, and embryos were processed for WMISH as previously described [23,25]. Primers used for probe generation are listed in Table S2; alternatively, probes were generated from plasmids received as gifts: *DKK1*, *WNT5a*, *PTCH2*, (Dr. Clifford Tabin); *FZD4* (Dr. Philippa Francis-West); *FGF8* (Dr. Eric Swindell); *PYST1*/*DUSP6* (Dr. Stephen Keyse). For all WMISH procedures, 3–5 embryos were examined for each gene per experiment and at least two independent experiments were performed.

### 2.6. RT-qPCR Validation of Transcriptome Data and Quantitation of LHX2 Overexpression/Knockdown Analysis

Further validation of the differential expression pattern of selected genes following 3 and 24 h of FGF treatment was performed via RT-qPCR. RNA was extracted from FGF2- or PBS-treated tissue 3 and 24 h after bead implantation. In order to minimize biological variation, 7–10 embryos from each treatment group were pooled. Primers used for RT-qPCR validation are listed in Table S2.

RT-qPCR was also used to detect changes in *SHH* and *PTCH2* expression following overexpression and knockdown of LHX2. For all samples, RNA extraction and on-column DNA digestion were performed using the RNeasy plus micro kit (Qiagen). The extracted RNA was converted to cDNA by reverse transcription using the iScript Advanced cDNA Synthesis kit (Bio-Rad, Irvine, CA, USA). Primers used are listed in Table S2.

The RT-qPCR experiments were performed using the SsoAdvanced Universal SYBR Green Supermix (Bio-Rad, Irvine, CA, USA) on the CFX96 ThermoCycler and analyzed using the CFX Manager 3.0 software (Bio-Rad). All RT-qPCR reactions were performed in triplicate for at least 2 independent experiments. Gene expression levels relative to a PBS-treated control were calculated using the 2^−ΔΔCT^ method after values were normalized to the housekeeping gene *PGK1*. The resulting fold changes are plotted with error bars representing the standard deviation. The significance (*p*-value) was calculated using the Student’s paired *t*-test.

### 2.7. Comparative Transcriptome Analysis

Integration of the RNA-seq and microarray data into R coding platforms facilitated categorization of genes according to their fold change and *p*-values then visualized by a volcano plot and table. We ranked the genes by dividing the fold change of each gene by their corresponding *p*-value. This narrowed down our target selection to LHX2.

### 2.8. Gene Overexpression and Knockdown via Electroporation

A plasmid encoding the mouse Lhx2 gene (pCGC-Lhx2; used at a final concentration of 1 µg/µL) or the translation-blocking anti-*LHX2* morpholino (1 mM) was injected into HH23 chicken embryo forelimbs around the FGF bead or into the ZPA. Confined micro-electroporation (CMEP) as previously described [22] was used to introduce the plasmid or morpholino into the limb cells surrounding the injection site. A β-actin promoter-driven red fluorescent protein (RFP) vector (pCAGGS-RFP) was used as an empty vector control. Sterile mineral oil is used in our syringe and needle to facilitate accurate DNA delivery. After injection, a small amount of oil is typically introduced to occlude the site of injection and confine the DNA to the target region.

The LHX2-GFP expression plasmid (pCGC-*Lhx2*) was received as a gift from Dr. Shubha Tole and used with the permission of Dr. Toshi Ohshima. The design and efficacy of the vector has been previously reported [26]. Representative data from three independent experiments is shown in the results section with number of embryos assayed included in the figure legends.

A translation-blocking anti-*LHX2* morpholino (GeneTools, LLC, Philomath, OR, USA) was designed against chicken *LHX2* (NCBI Reference Sequence: NM_204889.1). A negative-control morpholino was generated from the anti-*LHX2* sequence incorporating 5 base mismatches to alter the sequence. A positive-control anti-*SHH* morpholino was also generated against chicken *SHH* (NCBI Reference Sequence: NM_204821.1) A 3′ carboxyfluorescein (green) tag was incorporated into the anti-c*LHX2* and anti-c*SHH* morpholinos, while a lissamine (red) tag was incorporated on the 3′ end of the negative-control morpholino (anti-c*LHX2*-5mis). The fluorescent tags were used to assess targeting of the injection and electroporation efficiency prior to harvesting embryos for WMISH or RT-qPCR analysis. Morpholino (MO) sequences are as follows: anti-c*SHH* MO: 5′-TTGTCAACAGCAGCATTTCGACCAT-3′; anti-c*LHX2* MO: 5′-ACAGGCTGTGGAAAAGCATCGCT-3′; anti-c*LHX2*-5mis: 5′-AgAGcCTGTcGAAAAcCATCGgT-3′. In the 5-base mismatch morpholino (anti-*cLHX2*-5mis), nucleotides in lower case specify the mismatches. All morpholinos were used at a concentration of 1 millimolar.

#### Controlling for Off-Target Effects Using Morpholinos

To control for target specificity, we used 3 previously described approaches [27,28], including: (1) The use of a mismatched negative-control morpholino that differed from the anti-target morpholino by 5 nucleotides; (2) comparison of phenotypic changes with published reports. Our LHX2 knockdown was consistent with published reports of *Lhx2* mutants [29,30]; (3) we used “rescue” experiments that showed that overexpression of *Lhx2* was able to recover the expression of its downstream target, *SHH*.

## 3. Results

### 3.1. FGF Can Maintain and Induce SHH Expression in the Posterior Limb Bud

*SHH* expression persists in the former ZPA domain following implantation of an FGF-laden bead. *SHH* is detectable as early as 3 h after application; at 24 h, robust *SHH* expression is also induced around the bead (non-ZPA domain) (Figure 1B). This spatial and temporal difference in *SHH* upregulation suggests that induction or reactivation of *SHH* expression outside the former ZPA domain (24 h) requires additional FGF-mediated factors besides those required to maintain *SHH* in the former ZPA domain (3 h). We analyzed the transcriptome along the posterior margin proximal to the ZPA 3 h after FGF application and surrounding the FGF-laden bead after 24 h to uncover molecules involved in FGF-mediated SHH expression (areas outlined by dotted lines in Figure 1).

### 3.2. Brief (3 h) FGF Exposure Affects Biological Processes Associated with Its Role in Gene Expression

**Figure 2:**
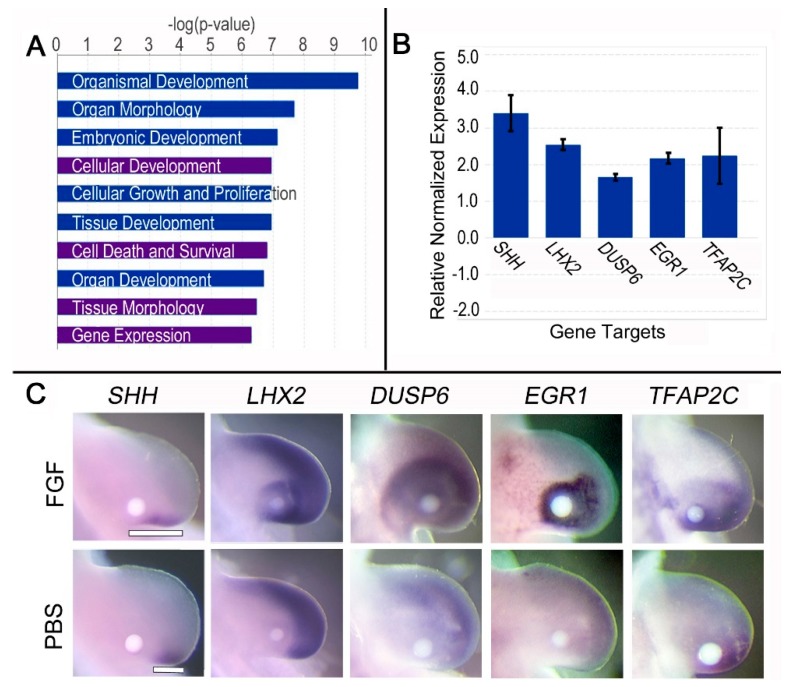
Brief (3 h) FGF treatment promotes transcription of early developmental genes and maintains *SHH* expression in the former ZPA domain. (**A**) Top pathways and biological processes affected 3 h after FGF treatment. Purple bars indicate top pathways affected that differed from the 3 h transcriptome analysis. (**B**) Fold change of selected targets treated with FGF for 3 h compared to PBS treatment via RT-qPCR (*p* < 0.05). The y-axis shows the relative normalized gene expression. Assays were performed in triplicate and at least two independent experiments were performed. Expression of each gene was compared to a PBS control (*p* < 0.05 for each comparison). (**C**) WMISH validation of selected targets regulated by FGF after 3 h exposure. Top panel shows upregulation of respective genes, while the bottom panel consists of PBS-treated limbs. n = 6 embryos per gene for FGF treatment and 3 embryos per gene for PBS. White bars highlight the proximal extension of the S*HH* expression domain in the FGF-treated limb.

We found 150 genes differentially regulated 3 h after FGF exposure by RNA-seq analysis, with 102 targets upregulated and 48 downregulated (*p* < 0.05). Four of the top biological processes affected by FGF exposure were identified in our 3 h dataset, but not our 24 h dataset: “Gene Expression”, “Cell death and survival”, “Cellular development”, and “Tissue morphology” (Figure 2A). This collection of biological processes was predicted to be downstream of FGF2 and FGF8, in addition to some other growth factors. Moreover, limb-related functions formed subsets of many of these biological processes (listed in Table S1).

Approximately 30% of the targets (n = 42 out of 150) were associated with “Gene Expression”, with 36 categorized as transcription/translation regulators. Transcription factors Early growth response 1 (*EGR1*), LIM Homeobox 2 (*LHX2*), and Transcription factor AP2 gamma (*TFAP2C*) were upregulated 12.5, 3.9, and 3.4-fold, respectively. Their regulation in response to 3 h FGF exposure was confirmed by WMISH and quantified by RT-qPCR (Figure 2B,C).

### 3.3. Brief (3 h) FGF Exposure Regulates Genes Involved in FGF Feedback Inhibition, Distalization, and SHH Expression

After 3 h of FGF treatment, Dual specificity phosphatase 6 (*DUSP6*) and Sprouty 2 (*SPRY2*), both negative regulators of FGF signaling, were upregulated 2.6-fold and 3.4-fold, respectively, consistent with FGF feedback inhibition. The upregulation of *DUSP6* was validated by WMISH and RT-qPCR (Figure 2B,C). Coupled with *DUSP6* and *SPRY2*, several other distally restricted genes were upregulated in the former ZPA domain 3 h after FGF exposure, including Bone morphogenetic protein 4 (*BMP4*; 1.8-fold). BMP4 plays a role in AER maintenance and regression and cooperates with SHH in determining digit identity [31,32]. In contrast, the proximally restricted Pre-B-cell leukemia homeobox 1 (*PBX1*) was downregulated 1.8-fold. *Pbx1*-deficient mice have malformations in proximal limb elements, suggesting a role in proximal limb development [33]. Downregulation of proximal markers by brief FGF exposure and the upregulation of distal factors support a distal respecification of the mid-proximal limb, which may be necessary for *SHH* expression.

Several targets known to be upstream of *SHH* expression, including Homeobox D9 [34,35], *ELF2* (a member of the ETS family), and *HAND2* (a basic helix–loop–helix transcription factor) were up-regulated 2.3-fold, 1.9-fold, and 1.6-fold, respectively. A complex interaction between HOX, PBX, ETS, and HAND2 has been implicated in the activation and localization of *SHH* expression to the ZPA [36]. These transcription factors coordinate their interactions on *SHH* expression through a conserved long range *cis*-regulatory module, the ZPA regulatory sequence (*ZRS*) [37]. *LHX2* and *TFAP2C* are two additional distally restricted transcription factors with potential binding sites in the *ZRS* that were upregulated after 3 h of FGF treatment. Our dataset revealed a 2.6-fold increase in *SHH* expression after 3 h of FGF exposure, which is supported by in situ and RT-qPCR data (Figure 2B,C).

### 3.4. Prolonged (24 h) FGF Exposure Affects Cell Processes Related to Organ and Organismal Development

**Figure 3:**
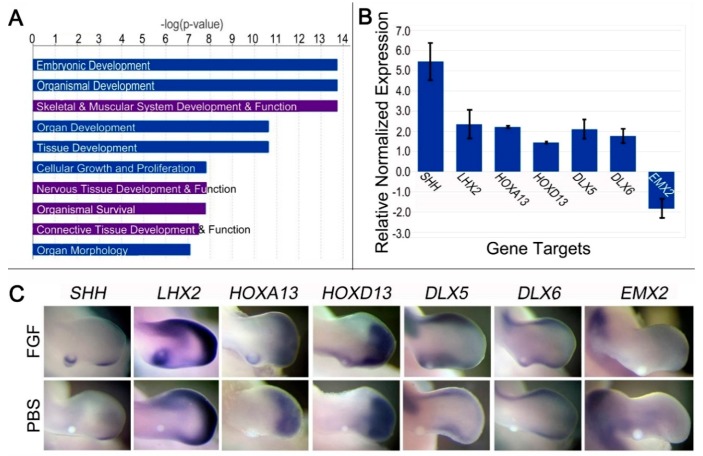
Prolonged (24 h) FGF treatment in the mid-proximal limb bud promotes distalization, supports processes involved in embryonic development, and leads to SHH induction. (**A**) Top pathways and biological processes affected by 24 h of FGF treatment. Purple bars indicate top pathways affected that differed from the 3 h transcriptome analysis. (**B**) RT-qPCR validation of FGF-mediated expression of target genes. The y-axis shows the relative normalized expression of selected target genes. Assays were performed in triplicate and at least two independent experiments were performed. Expression of each gene was compared to the same region on the contralateral limb buds (*p* < 0.05 for each comparison). (**C**) WMISH of distally restricted transcription factors confirm their regulation in response to 24 h of FGF exposure. Top panel shows upregulation/downregulation in response to ectopic FGF when compared to a PBS-treated limb (control; bottom panel). n = 6 embryos per gene for FGF treatment and 3 embryos per gene for PBS. Note that *EMX2* is strongly expressed proximally, but has a weak distal expression that was reduced by FGF treatment after 24 h.

DNA microarray analysis identified 3434 differentially regulated targets after 24 h of FGF exposure (*p* < 0.05). In addition to *SHH*, 2152 targets were upregulated, while 1281 were downregulated. Of the differentially expressed targets, 1085 mapped to IPA-curated genes. IPA revealed the top 10 biological processes were related to growth and development. Four processes were found at 24 h that were not present at 3 h: “Skeletal Muscle System Development and Function”, “Nervous Tissue Development and Function”, “Organismal Survival”, and “Connective Tissue Development and Function” (Figure 3A). IPA also predicted that the molecules, functions, and biological processes identified in the microarray data were downstream of FGF2, FGF4, and FGF8. Several limb-related functions form subsets of the biological processes mined by IPA (Table S1).

Of note, an abundance of differentially regulated genes encoding Wnt signaling proteins were detected (n = 46). Wnt proteins are involved in limb initiation, outgrowth, cell migration, cell differentiation, and patterning [38,39]. This led us to question whether a Wnt pathway might be involved in the upregulation of *SHH*. However, overexpression of the highest upregulated Wnt protein (*WNT5a*; 1.6-fold) in the posterior limb bud did not lead to ectopic *SHH* expression (Figure S1). 

### 3.5. Prolonged FGF (24 h) Regulates Genes Associated with Dedifferentiation, Distalization, and SHH Regulation

Several transcription factors associated with cell dedifferentiation were upregulated in response to prolonged FGF exposure: Spalt-Like Transcription Factor 4 (*SALL4*; 1.8-fold), Muscle segment homeobox genes 1 and 2 (*MSX1*; 4.4-fold and *MSX2*; 3.0-fold), *LHX2* (5.6-fold), and Matrix metallopeptidase 11 and 17 (*MMP11* and *MMP17*, each 1.4-fold). FGF treatment also downregulated members of the collagen family, which are associated with limb differentiation and chondrocyte maturation (*COL12A1*, *COL6A1*, *COL6A3*, *COL21A1*; >1.5-fold). Dedifferentiation of the mid-proximal limb bud may be a crucial step in reprogramming this region to express *SHH*. Alternatively, FGF may be functioning to prevent differentiation and promote the maintenance of cells in an undifferentiated state. Another step could be the distal respecification of the mid-proximal limb supported by the upregulation of several distally restricted genes, including *HOXA13* (13.0-fold), *HOXD13* (1.7-fold), *DLX5* (2.2-fold), *DLX6* (3.6-fold), and *LHX2* (5.6-fold) (Figure 3B,C); while proximally restricted genes such as Empty spiracles homeobox 2 (*EMX2*) and *MEIS2* were downregulated 1.7- and 1.5-fold, respectively. Downregulation of *EMX2* was validated by WMISH and RT-qPCR (Figure 3B,C). Together, the data suggest that dedifferentiation and the distal respecification of the mid-proximal limb bud mesoderm play a role in FGF-mediated *SHH* expression.

After 24 h of FGF exposure, *SHH* is upregulated 2.1-fold around the FGF bead. This correlated with RT-qPCR data (upregulated 5.5-fold) (Figure 3C). Genes associated with *SHH* expression include the distally restricted *HOXA13*, *HOXD13*, and *LHX2*. Other upregulated *SHH*-associated genes include *HOXA10* (1.4-fold), *HOXA11* (1.7-fold), Gap junction protein alpha 1 (*GJA1*; 1.3-fold), *ETS2* (1.3-fold), and *ETV5* (1.1-fold). Although the fold change for *ETV5* might be less than typical cutoffs, it has a *p* value < 0.05 and an established functional role in linking FGF signaling to the *ZRS* and *Shh* expression [40,41]. The *SHH* antagonist Aristaless-like homeobox 4 (*ALX4*) was downregulated 1.7-fold. ETS2, ETV5, and ALX4 contribute to the spatial localization of *SHH* expression [41,42,43]. Notably, our 24 h dataset includes upregulation of factors downstream of SHH signaling such as *PTCH1* (1.8-fold) and *PTCH2* (1.1-fold). Because the 24 h transcriptome may include a number of downstream targets of SHH, we compared it with the 3 h transcriptome to highlight common genes most likely to be upstream of *SHH*.

### 3.6. Common Pathways Accentuated by Comparative Transcriptome Analysis

**Figure 4:**
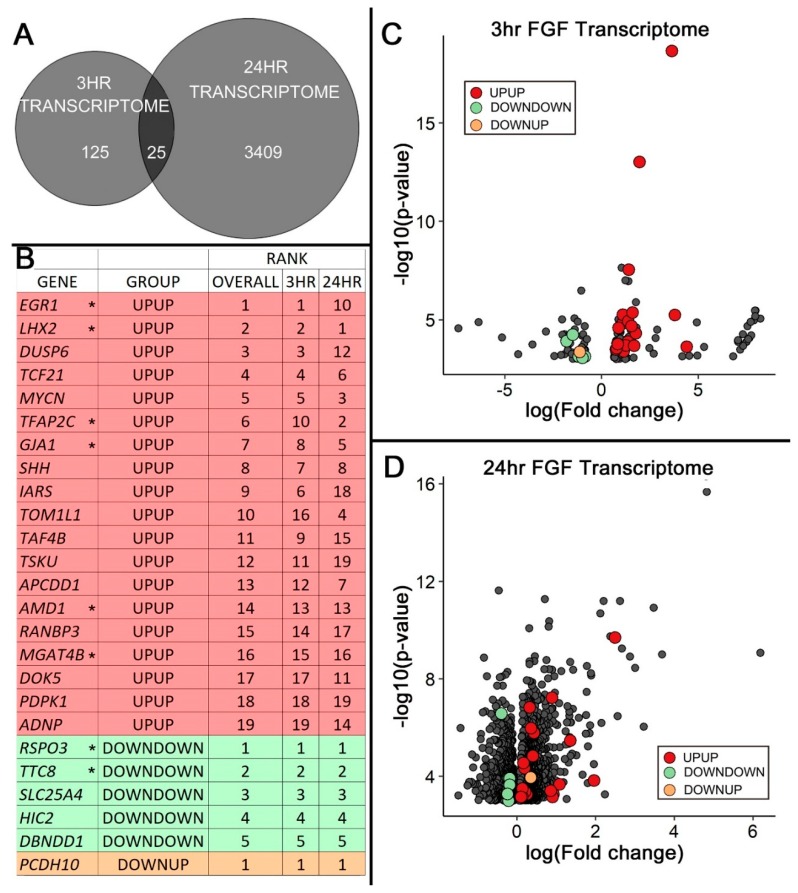
Common transcripts regulated by FGF2 during maintenance and induction of *SHH* expression are likely candidates for FGF-mediated SHH expression. (**A**) The number of FGF-regulated transcripts common to both 3 and 24 h datasets is shown in the overlapping region of the Venn diagram (n = 25). (**B**) Table showing the 25 common targets from the 3 and 24 h transcriptome that are likely candidates for FGF-mediated SHH expression. Targets are ranked based on differential expression (fold change) divided by significance (*p*-value). Asterisks (*) denote genes associated to *SHH* expression/function. Genes upregulated at both 3 and 24 h are designated with Group ID “UPUP”: red (n = 19). Genes downregulated at both time points are designated “DOWNDOWN”: green (n = 5), while genes downregulated at 3 h and upregulated at 24 h are designated “DOWNUP”: orange (n = 1). (**C**) Distribution (by log10 *p*-value and log2 fold change) of the 150 genes differentially regulated after 3 h FGF treatment, with the 25 common targets highlighted in color (*p* < 0.05). (**D**) Distribution (by log10 *p*-value and log2 fold change) of the 3434 genes differentially regulated by 24 h FGF treatment, with the 25 common targets highlighted in color (*p* < 0.05).

Six of the top IPA-curated pathways affected by FGF treatment were common between the 3 and 24 h datasets, including “Embryonic/Organismal Development” and “Growth and Proliferation”: hallmarks of FGF signaling (Figure 2A and Figure 3A). We also detected mutual limb-related functions associated with limb development and digit morphogenesis (see Table S1). Twenty-five genes were common to both transcriptomes, with 19 being upregulated, five downregulated, and one gene downregulated at 3 h but upregulated at 24 h (Figure 4).

#### 3.6.1. Common Targets Involved in Wnt Signaling

We identified five genes associated with the Wnt pathway within the shared 25 FGF-regulated genes. *APCDD1* [44] and *RANBP3* [45] are Wnt signaling inhibitors that were upregulated by FGF, while R-spondin 3 (*RSPO3*), a secreted ligand that binds cell-surface receptors and activates Wnt/β-catenin or Wnt/planar cell polarity signaling [46,47], was downregulated. One downstream target of Wnt signaling, SNAI2, was upregulated, while another, *TUSC3* [48], was downregulated. Taken together, these data support a role for Fgf in the complicated regulation of Wnt signaling during limb development [39,49].

#### 3.6.2. Common Targets Associated with SHH Expression

Of the 25 common targets, eight relate to *SHH* expression, function, or signaling (highlighted by asterisks in Figure 4B). Four genes associated with *SHH* expression were upregulated: *EGR1*, *GJA1* (also known as Connexin 43), *LHX2*, and *TFAP2C*. *TTC8*, which is associated with ciliary-associated GLI-Kruppel family member (GLI) processing, was downregulated. Collectively, the regulation of these genes is expected to enhance SHH function/signaling. Paradoxically, *RSPO3*, a gene associated with *SHH* upregulation, was downregulated. *AMD1* and *MGAT4B* were both upregulated and are decreased in Shh-deficient mice [50]. They could therefore be downstream of Shh signaling. Neither *AMD1* nor *MGAT4B* has been well-characterized, however, and they require further investigation.

### 3.7. LHX2 as an Intermediate in FGF-Regulated SHH Expression 

**Figure 5:**
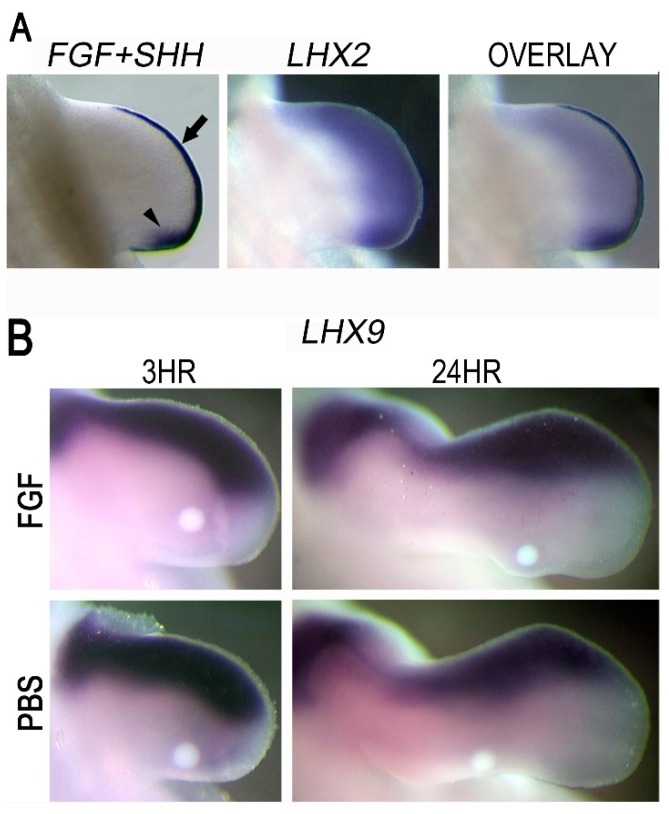
The *LHX2* expression pattern supports a role for maintaining distal posterior *SHH* expression during progressive limb outgrowth. (**A**) Left panel shows endogenous *FGF8* (arrow) and *SHH* (arrowhead) expression in a HH23 limb bud. Middle panel shows endogenous *LHX2* expression. Right panel is a composite of all three expression patterns. *LHX2* expression is restricted to the distal mesoderm in the developing limb subjacent to the *FGF8*-expressing AER. Importantly, *SHH* is expressed only within the posterior boundary of the *LHX2* expression domain. The overlapping expression pattern is consistent with a role for LHX2 in facilitating SHH expression in the developing limb. (**B**) After 3 and 24 h of FGF2 exposure, no appreciable ectopic *LHX9* expression is observed (n = 3 embryos per treatment group for each time point). PBS beads were used as controls. Note that endogenous *LHX9* expression is restricted to the anterior mesoderm.

Our screening identified LHX2 as a candidate for mediating FGF regulation of SHH. In the limb, *LHX2* is robustly upregulated by FGF following 3 and 24 h of exposure (Figure 2 and Figure 3). Additionally, *LHX2* is distally restricted subjacent to the AER overlapping the ZPA (Figure 5A). Since Lhx9 appears to play a redundant role in regulating *Shh* expression in mice, we evaluated its expression pattern in the chicken model. In chicken wings, *LHX9* is restricted to the anterior margin of the developing limb mesoderm and does not overlap the ZPA. Moreover, *LHX9* was not significantly upregulated in our transcriptome data and not convincingly upregulated around applied FGF beads (Figure 5B). Thus, in chickens, LHX2, but not LHX9, could function as an intermediate in the FGF-to-SHH pathway. 

**Figure 6:**
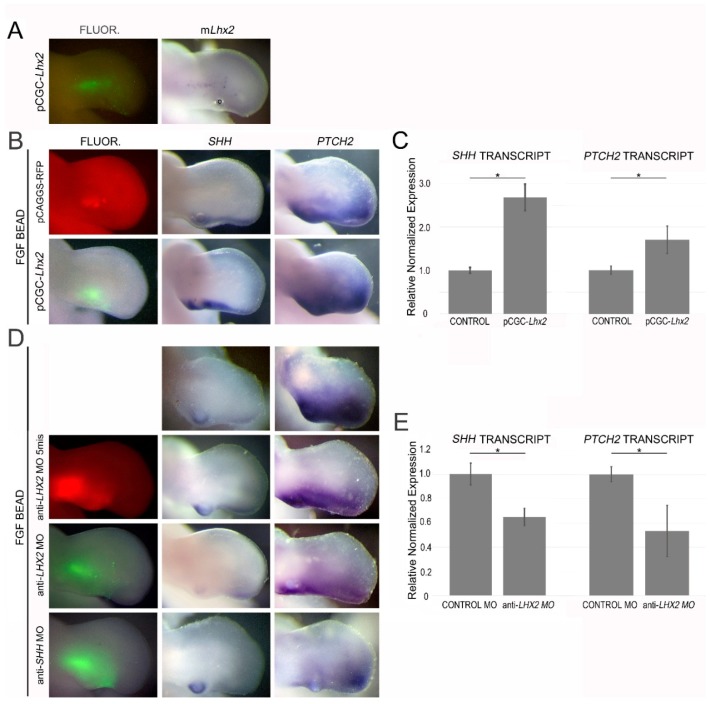
LHX2 regulates SHH expression and function. (**A**) Fluorescence (FLUOR.) and whole mount in situ hybridization (WMISH) pictures confirming ectopic mouse *Lhx2* following electroporation of an Lhx2 expression vector containing a GFP reporter (pCGC-*Lhx2*). “o” is used to identify the oil bubble which is occasionally present after the injection of a plasmid. (**B**) Increasing the level of local LHX2 by electroporation of pCGC-*Lhx2* around an FGF bead leads to an increase in the expression of *SHH* (n = 12) and its downstream target *PTCH2* (n = 8) when compared to an empty vector control (pCAGGS-red fluorescent protein (RFP)). (**C**) RT-qPCR data from two independent experiments reveals a 2.7-fold upregulation of FGF-induced *SHH* and a 1.7-fold upregulation of *PTCH2* transcripts in the presence of additional LHX2 when compared to FGF alone (*p* < 0.05). Tissue samples from 7–10 embryos were pooled for each treatment group and assayed in triplicate. (**D**) Transfection of an anti-*LHX2* morpholino decreases FGF-induced *SHH* expression (third row; n = 7) when compared to limbs treated with FGF beads only (top row; n = 5) or FGF with a negative control (five-base mismatch anti-*LHX2* MO; second row; n = 5). *PTCH2* expression was also decreased following treatment with the anti-*LHX2* morpholino (n = 10) and mimics the *PTCH2* reduction observed with a knockdown of SHH using an anti-*SHH* MO (n = 5). *SHH* expression is not affected by the electroporation of an anti-*SHH* morpholino designed to block translation of *SHH* transcript (bottom panel; n = 3). (**E**) Following a knockdown of LHX2, *SHH* and *PTCH2* transcript levels were reduced by 35% and 53%, respectively, when compared to the anti-*LHX2* MO five-base mismatch control (*p* < 0.05). Tissue samples from 7–10 embryos were pooled for each treatment group and assayed in triplicate for two independent RT-qPCR runs. * *p* < 0.05.

**Figure 7:**
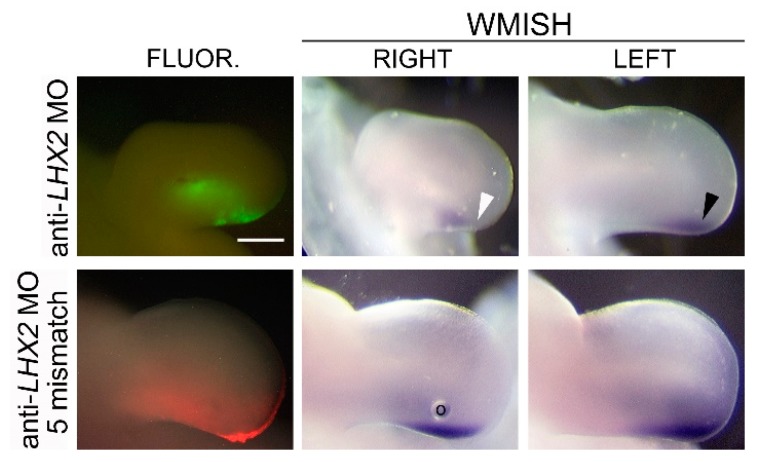
LHX2 knockdown at the ZPA decreases *SHH* expression. Twenty-four hours after electroporation of anti-*LHX2* MO at the ZPA, there is reduced *SHH* expression and retarded limb outgrowth when compared to the contralateral limb (LEFT) and to the negative control (anti-*LHX2* MO five-base mismatch). Reduced *SHH* expression and retarded limb outgrowth were observed in nine out of 10 embryos from two independent experiments. Please note the oil bubble “o” in the bottom row (middle panel). The images of the left contralateral control limbs have been reversed for comparison. Scalebar = 0.25 inches.

To determine the role of LHX2 in FGF-regulated *SHH* expression, we electroporated a mouse Lhx2 expression vector into the posterior limb mesoderm adjacent to an FGF- or PBS-soaked bead. Lhx2 was not sufficient to induce *SHH* independently. However, when combined with FGF, Lhx2 increased the expression of *SHH* (2.7-fold by RT-qPCR) and *PTCH2*, a downstream target of SHH signaling (Figure 6). We also examined the loss of LHX2 function using an anti-*LHX2* morpholino (anti-c*LHX2* MO) designed to block translation of the chicken *LHX2* transcript. Electroporation of this morpholino around an FGF bead implant resulted in decreased *SHH* expression (35% by RT-qPCR) and a decrease in *PTCH2*. Targeted SHH knockdown gives a similar decrease in *PTCH2* expression (Figure 6). Additionally, a decrease in LHX2 at the ZPA resulted in decreased limb outgrowth and reduced endogenous *SHH* expression after 24 h (Figure 7). This is consistent with a previous report that suggested that competitive inhibition of Lhx2 activity inhibits limb outgrowth in chicken as well as interfering with the expression of distally restricted genes such as *SHH* [29].

## 4. Discussion

One of the roles of apical ectodermal ridge (AER)-secreted FGFs is the maintenance of *SHH* expression in the subjacent ZPA during limb outgrowth [15]. We were able to maintain the expression of *SHH* in the former ZPA domain by application of FGF proximal to the ZPA. Persistent *SHH* expression was evident within as little as 3 h. FGF was also able to induce *SHH* in non-ZPA domain-related mesoderm, although this required prolonged exposure (24 h) (Figure 1). Cells in the population responding to prolonged FGF are likely to include some prior SHH-expressing cells. Most of the prior SHH-expressing cells, however, are present within the autopod and prospective digits at a comparable stage of development [9]. Irrespective of their prior history, induction or reactivation of cells in the non-ZPA domain requires prolonged FGF exposure to induce *SHH* expression and a mechanism disparate from that of cells residing within the former ZPA domain. By comparing transcriptome data from these two populations of *SHH*-expressing cells, we were able to identify 25 common genes, including 19 synexpressed with *SHH* and five downregulated.

### 4.1. FGF Regulates Genes that Support SHH Expression and Signaling/Function

Eight of the 25 common targets differentially regulated by FGF exposure were associated with *SHH* expression, signaling, or function (Figure 4). Consistent with our data, EGR1 has been reported to act downstream of FGF signaling [51,52]. Additionally, it serves as a direct transcriptional regulator of *SHH* in glioma cells [53]. In the limb, EGR1 participates in FGF-induced tendon differentiation [52], but its role in FGF-mediated *Shh* expression has yet to be evaluated. FGF upregulated *GJA1*, which is a gene that encodes Connexin 43, a gap junction protein. Gap junction proteins have been reported to relay FGF signals to neighboring cells; moreover, the conditional knockout of Connexin 43 in mice leads to reduced *Shh* expression, limb truncation, and patterning defects [54,55]. Thus, an increase in *GJA1* in both of our datasets suggests that FGF supports *SHH* expression by upregulating intercellular communication. FGF also upregulated *TFAP2C.* TFAP2C is an activating, enhancer-binding protein-2 (AP2) transcription factor expressed subjacent to the AER in normal limbs (Figure 2C). The ZPA regulatory sequence (*ZRS*) contains a potential AP2 binding site. Interestingly, a mutation generating an extra AP2 binding site in the *ZRS* is associated with anterior ectopic *SHH* expression and preaxial polydactyly [56]. Although its expression pattern and role in misregulation of the *ZRS* are intriguing, a role for AP2 transcription factors in normal *SHH* expression has yet to be determined. 

Tetratricopeptide Repeat Domain 8 (TTC8), also known as Bardet Biedl syndrome 8 (BBS8), was downregulated in our analysis. BBS8 is part of the stable core protein complex of cilia involved with Smoothened (Smo) ciliary trafficking, which processes Gli2 and Gli3 [57,58]. Processed GLI transcription factors are truncated and repress SHH targets [59]. In humans, TTC8 mutations are associated with pre- and post-axial polydactyly, consistent with activation of the hedgehog pathway [60,61]. Tayeh and colleagues showed that loss of BBS function in zebrafish increased fin/limb expression of *SHH* [62]. Thus, the regulation of TTC8 by FGF offers a mechanism to enhance the activation of SHH that warrants further investigation.

R-spondin 3 (RSPO3) is downregulated, but potentially functions upstream of SHH. A *Rspo2*/*Rspo3* double mutant showed more severe limb defects than either single mutant with the most anterior and posterior digits missing, leaving three shortened middle digits [46]. The fact that posterior elements in the forelimb are lost in this double mutant correlates well with the phenotype of *SHH-*deficient limbs [7,8,63] and suggests that R-spondin genes could affect *SHH* expression/function. No limb expression patterns were found for *AMD1* and *MGAT4B*, but our data indicate that they are upregulated by FGF signaling. Additionally, *Amd1* and the Mgat4b paralog *Mgat4a* are reported to be decreased in the limbs of Shh-deficient mice [50], indicating that they may be downstream of SHH signaling. 

### 4.2. FGF Regulates Factors that Localize SHH Expression

The *ZRS* is a conserved *cis*-acting regulatory element responsible for limb-specific *SHH* expression [7,37,64,65]. The *ZRS* houses multiple binding sites for the ETS/ETV family of transcription factors. At 3 h, *ELF2* (E74-like ETS transcription factor 2) and *ETV6* were upregulated, while *ETS2* and *ETV5* were upregulated at 24 h. Lettice et al. reported that a balance between occupancy of the ETS/ETV binding sites within the *ZRS* contributes to the expression and location of *Shh* in the limb bud [42]. The upregulation of members of the ETS family in our data supports the notion that FGF regulates ETS/ETV transcription factors to modify *SHH* expression in the developing limb and highlights the possibility that family members other than ETS2 and ETV4/5 [41,42] may be involved. 

The TAATTA binding motif for LHX2 [66] is found in the *ZRS* and is conserved across 16 vertebrate species including the human, mouse, and chicken [67]. Of note, other Hox proteins share this binding motif and 5′ Hox genes have been reported to bind the ZRS [33]; therefore, LHX2 binding to this region needs to be confirmed.

### 4.3. LHX2, but Not LHX9, Regulates FGF-Mediated SHH Expression during Chicken Limb Development

Reports have suggested that LHX2 and a homolog LHX9 may be functionally redundant due to their overlapping expression patterns [30,68,69]. Simultaneous knockout of *Lhx2* and *Lhx9* causes a marked reduction in *Shh* expression and altered limb growth and patterning characterized by oligodactyly, loss of digit morphology, and a shortened limb [30]. In chickens, *LHX2* is expressed in the distal mesoderm subjacent to the AER in a pattern that overlaps the ZPA, while *LHX9* is restricted to the anterior and distal rim of the limb mesoderm distant from the ZPA (Figure 5) [29,70]. Unlike the mouse model, where an *Lhx2*/*Lhx9* double knockout was necessary to perturb limb outgrowth and *Shh* expression, Rodriguez-Esteban and coworkers showed that a retroviral LHX2-targeted repressor caused limb truncations [29]. We further show that targeted knockdown of LHX2 within the ZPA is sufficient to decrease *SHH* expression and disrupt limb outgrowth (Figure 7). These collective findings indicate a species-specific difference in the function of Lhx family members, but highlight Lhx2 as a common mediator of *Shh* expression. 

Although the pattern of *Lhx2* and *Lhx9* expression in mice is consistent with regulation by FGF, Tzchori et al. suggested that Fgf4/8 did not control their expression [30]. In contrast, Yang and colleagues showed that Fgf signaling was required for *Lhx9* expression in mouse limb explants [71]. In chicken, we demonstrate by transcriptome, WMISH, and RT-qPCR that FGF robustly upregulates *LHX2*, but not *LHX9*. *LHX2* overexpression or knockdown in the context of ectopic FGF-bead application also resulted in a robust increase or decrease in *SHH* expression, respectively. Our data indicate that LHX2 is a target of FGF signaling and an intermediate in the FGF to SHH regulatory loop. 

Interestingly, Tzchori and colleagues demonstrated that Ldb1, a cofactor of LIM transcription factors, was required for FGF-mediated induction of *Shh*. *Ldb1* is ubiquitously expressed in the limb and is known to associate with a variety of LIM-domain genes [72,73]. The widespread expression of Ldb1 and its required presence to permit Fgf-mediated *Shh* expression points to a cofactor, such as Lhx2, as an intermediate in upregulating *Shh*.

### 4.4. LHX2 as a Competency Factor for SHH Expression in the Limb

Our data corroborates other reports that indicate LHX2 is necessary for *SHH* expression [30]. Additionally, *LHX2* expression overlaps the ZPA and the proximal extent of *LHX2* expression corresponds to the proximal boundary of the ZPA; beyond this boundary, *SHH* expression wanes. Taken together, LHX2 is likely a competence factor for *SHH*, keeping its expression juxtaposed to the AER during progressive limb outgrowth. Other known competence factors such as *HoxB8* [74], *Hox9* paralogs [34], and *Hand2* [75,76] display expression domains larger than, but inclusive of, the ZPA to permit *SHH* expression, indicating a collective cooperation among competence factors. 

Ectopic apical *Shh* expression subjacent to the AER in an *Lhx2*-like pattern has been reported [77,78]. Interestingly, in both reports, there was a reduction in *Gli3*. *Gli3* is expressed throughout the limb, except within the distal posterior mesoderm [79], and together with Alx4 [43], Twist [80], and the Etv proteins [40,80], plays a role in restricting *Shh* expression to this limited posterior domain. Reduction in the repressive activity of Gli3 results in the anterior expansion of Hand2, while *Lhx2* expression in *Gli3*-deficient limb buds remains unchanged [81]. The ectopic apical *SHH* expression pattern overlapped the expanded *Hand2* domain, but was more distally restricted, suggesting that a distally restricted factor, such as Lhx2, was required. We suspect that Lhx2 is a transcription partner with Hand2 and other competency factors that function to regulate and maintain *Shh* transcription. 

In conclusion, we identified a common set of genes regulated by FGF with potential to function as intermediates in limb-related FGF-mediated *SHH* expression. Additionally, we have extended the role of LHX2 from previous reports, providing evidence that LHX2 mediates the FGF-to-SHH regulatory loop during limb development.

## Supplementary Materials

The Supplementary Materials are available online at http://www.mdpi.com/2221-3759/6/2/13/s1.
