# Supplementary figures and images for: LHX2 Mediates the FGF-to-SHH Regulatory Loop during Limb Development
Source: J Dev Biol. 2018 Jun 15;6(2):13. doi: 10.3390/jdb6020013 (PMC6027391; doi:10.3390/jdb6020013)

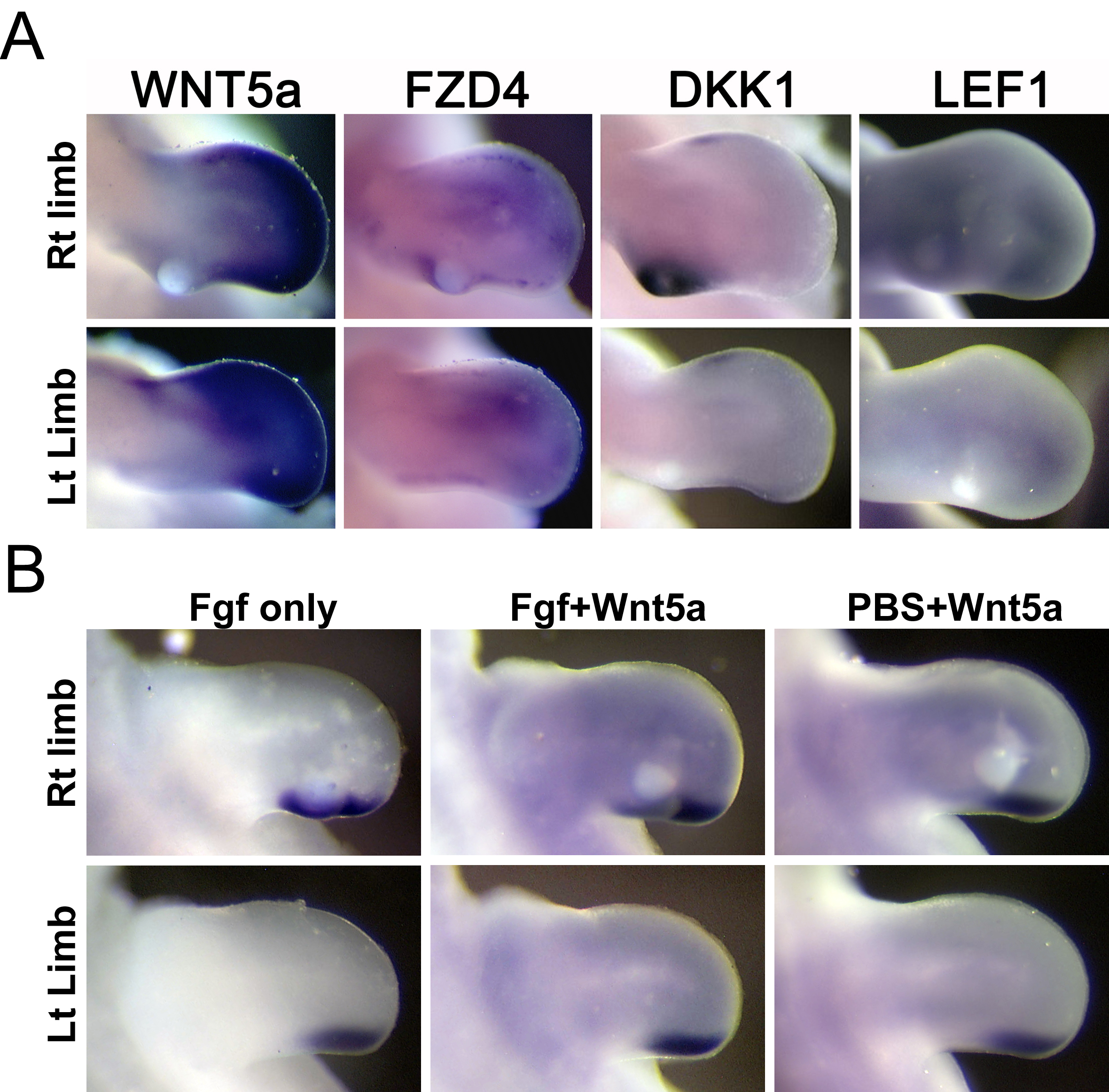

Supplement: Supplementary file 1 [file jdb-06-00013-s001.zip › supplementary data/Fig S1 _JDB.jpg]
